# Dietary intake of inorganic phosphorus has a stronger influence on vascular-endothelium function than organic phosphorus

**DOI:** 10.3164/jcbn.17-97

**Published:** 2018-01-19

**Authors:** Hiromi Kawamura, Sarasa Tanaka, Yuri Ota, Sumire Endo, Mariko Tani, Midori Ishitani, Motoyoshi Sakaue, Mikiko Ito

**Affiliations:** 1 Graduate School of Human Science and Environment, University of Hyogo, 1-1-12 Shinzaike-Honcho, Himeji, Hyogo 670-0092, Japan; 2 School of Human Science and Environment, University of Hyogo, 1-1-12 Shinzaike-Honcho, Himeji, Hyogo 670-0092, Japan

**Keywords:** inorganic phosphorus, endothelial function, food additive, flow mediated dilation

## Abstract

Phosphorus management through dietetic therapy is vital for the prevention of cardiovascular disease in chronic kidney disease patients. There are two main sources of phosphorus in the diet, organic phosphorus from protein and inorganic phosphorus from food additives. The adverse effects of high phosphorus intake on vascular-endothelium function have been reported; however, the differences in the effects of organic phosphorus versus inorganic phosphorus are not clear. In this study, we examined an acute effect of these high phosphorus meals intake on vascular-endothelium function. This was a randomized, double-blind, cross-over test study design targeting healthy young men. We conducted a food intake test using two test meals, one high in organic phosphorus from organic food sources, and one high in inorganic phosphorus from food additives. Endothelium-dependent vasodilation, phosphorus and calcium in the urine and blood, and phosphorus-related hormones were measured preprandial to 120 min postprandial. The results showed higher serum and urine phosphorus values after the high inorganic phosphorus meal, and a significant reduction in endothelium-dependent vasodilation at 30 min postprandial. These findings are evidence that inorganic phosphorus has a stronger influence on vascular-endothelium function than organic phosphorus.

## Introduction

Elevated serum phosphorus (P) levels have been associated with cardiovascular disease (CVD) and cardiovascular mortality in chronic kidney disease (CKD) patients.^(1–4)^ Serum P level is mainly regulated by 1,25(OH)_2_D, a parathyroid hormone (PTH), and fibroblast growth factor 23 (FGF23), and is maintained at 2.5–4.5 mg/dl in healthy populations.^(5–7)^ The major cause of elevated serum P is excessive P intake under the condition of kidney dysfunction.

P intake from meals is classified into organic P from natural foods and inorganic P from food additives such as pH control agents, coagulants and stabilizers. In healthy people, the rate of absorption of organic P is about 40–60% and that of inorganic P is 90% or more.^(8,9)^ Foods high in protein, such as meat, fish, and dairy products, are major sources of organic P, and it is rare for a P deficiency to develop with the usual dietary pattern of recent years. Rather, there are problems with excessive P uptake since it is used abundantly as an additive in processed foods. When renal function is normal, excessive P is excreted in the urine. However, since the renal function of dialysis patients is inadequate, excretion of P is insufficient, and hyperphosphatemia can easily develop.^(8,10)^

Hyperphosphatemia has been associated with arteriosclerosis and vascular calcification, and is an important factor in the onset of CVD in dialysis patients.^(1–4)^ Therefore, P management through dietetic therapy is important for dialysis patients.^(11)^ However, the high amounts of inorganic P found in food additives make P management difficult. In addition, there are no standardized criteria for inorganic P on food packaging labels in Japan and food manufacturers are under no obligation to provide this information, making it very difficult to ascertain the amount of P intake.

The dysfunction of vascular endothelial cells is an important effect of hyperphosphatemia.^(12)^ Vascular endothelial cells exist in the innermost layer of a vessel, and emit various substances essential to vessel operation, such as nitric oxide (NO), endothelin, C-type natriuretic peptide, and prostaglandin I_2_.^(13)^ Moreover, NO controls the cohesion and flocculation of thromboplastids, functions that protect vessels by preventing blood coagulation.^(12)^ It has been reported that a high P state causes an incremental increase in oxidative stress, a reduction in NO yield in systemic endotheliocytes, and induces an apoptotic state in CKD patients.^(14–16)^ When damage to endotheliocytes continues over the long term, the protective effect of a vessel fails and CVD due to arteriosclerosis develops.

A previous report investigated the influence of transient P intake by studying the effects of low and high P loading (meals with 400 and 1,200 mg, respectively) on vascular-endothelium function in healthy men using a P loading test for inorganic P.^(17)^ The results showed serum P levels were elevated after a high P intake, and serum P level and vascular-endothelium function had a negative relationship. These results are evidence that P from oral intake increases serum P concentration and affects vascular-endothelium function.

In this study, we hypothesized that inorganic P has a stronger influence on vascular-endothelium function than organic P and is connected with CVD. We investigated the differences in phosphate metabolism and the effects of consuming meals high in organic P and inorganic P on vascular-endothelium function.

## Materials and Methods

### Subjects

We conducted a food intake test for organic and inorganic P in ten healthy young men without hypertony, smoking history, present illness, or prescription history of vascular-endothelium function-related drugs or antioxidation supplements. The study protocols were approved by the Ethics Committee of the University of Hyogo. Signed written informed consent was obtained from all study subjects before participation. We finally chose six subjects, except men who exceeded standard level of preprandial serum P levels, FGF23 or endothelium-dependent vasodilation. The preprandial characteristics of the six subjects are shown in Table 1. This intake test did same six subjects eat both meals with a 7-day break in between.

Measurements of stature, weight, percent body fat, lean body mass, and body mass index (BMI) were taken using a digital stature meter AD-6400 (A&D Company, Ltd. Tokyo, Japan) or InBody720 Body Composition Analyzer (InBody Japan Inc., Tokyo, Japan). Measurements of blood pressure and pulse were performed using a fully automatic sphygmomanometer TM-265 (A&D Company, Ltd.) before the first food intake test.

### Study design

**Fig. 1:**
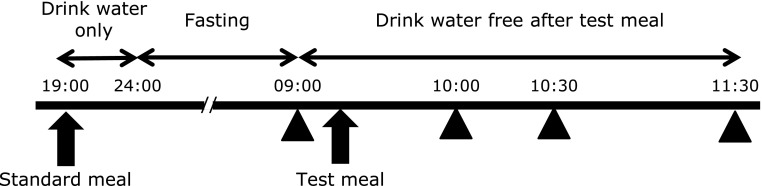
Study schema. On the day before the test, all subjects ate a standard meal at 19:00–20:00 and were asked to consume only water after eating. The subjects came to the laboratory in a fasted state and were allowed to drink water after the test meal. We measured %FMD and collected blood and urine samples (▲) at four time points: preprandial (0 min) and 30, 60 and 120 min postprandial. We also took anthropometric measurements and blood pressure at 0 min for the first meal only.

The study used a randomized, double-blind, crossover design on two different days separated by at least 7 days. The protocol is illustrated diagrammatically in Fig. 1. In order to eliminate various factors that could affect the measurements, dinner on the day preceding the food intake test for all subjects was a standard meal eaten from 19:00 to 20:00 and then only water was consumed until 24:00. The standard dinner consisted of 755 kcal with 16.1 g of protein, 163 mg of calcium (Ca) and 180 mg of P.

The subjects came to the laboratory at 08:30 after fasting from 24:00 the previous night and rested in a seated position for about 30 min in a room maintained at 25°C. The two test meals were served in random order. The food intake test was completed within 15 min, and subjects could drink freely thereafter. Measurements were performed for each test meal preprandial (0 min) and postprandial (30, 60 and 120 min). Measurements for body composition elements, blood pressure, and pulse were taken at 0 min only for the first meal. Vascular-endothelium function was measured as described below, and urine and blood were collected at all four time points.

The composition of the test meals is shown in Table 2. The high organic P meal containing organic P (1,200 mg) was prepared using natural food sources. The high inorganic P meal contained inorganic P from food additives (1,000 mg) and organic P (199.7 mg) from natural food sources. Nutritional information values were computed using the Standard Tables of Food Composition in japan 2010. Both test meals consisted of approximately 860 kcal with 31 g of protein, 1,200 mg of P (both organic and inorganic), and 490 mg of Ca. The P loading amount was chosen with reference to a preceding study.^(17)^ The upper limit (3,000 mg/day) according to the Japanese Dietary Reference Intake 2015 edition was taken into consideration as an amount which does not cause harm to health, and the appropriate P load was determined to be 1,200 mg.

### Biochemical determination

Blood samples were collected from the median cubital vein at four time points: preprandial (0 min), and 30, 60 and 120 min postprandial. P, Ca, 1,25(OH)_2_D, and intact PTH were measured at all four time points. Ionized Ca, high density lipoprotein cholesterol (HDL-C), low density lipoprotein cholesterol (LDL-C), triglycerides (TG), uric acid (UA), sodium (Na), chloride (Cl), and potassium (K) were measured at three points (0–60 min postprandial). All blood test measurements were performed by Hyogo Clinical Laboratory Corporation (Hyogo, Japan). Blood glucose level was measured at all four time points using the Glutest Neo Super blood glucose meter (Sanwa Kagaku Kenkyusyo Co., Ltd., Aichi, Japan) at the time a blood sample was collected. Serum FGF23 concentration was measured using FGF-23 ELISA Kit (Kainos Laboratories, Inc., Tokyo, Japan).

Urine was also collected at all four time points: preprandial (0 min) and 30, 60 and 120 min postprandial. Urine P, Ca and creatinine were measured at all four points. Urine pH, protein, sugar, urobilinogen, occult blood, bilirubin, and ketone bodies were measured at 0 min. All urine test measurements were performed by Hyogo Clinical Laboratory Corporation. Creatinine corrections were calculated for urine P and Ca concentrations in order to eliminate the influence of water intake or sweating.

### Measurement of endothelium-dependent vasodilation

We evaluated endothelial function by measuring flow mediated dilation (FMD; UNEXEF18VG, UNEX Corporation, Aichi, Japan), according to previously published guidelines.^(18,19)^ %FMD was measured at four time points: preprandial (0 min) and 30, 60 and 120 min postprandial. If vascular-endothelium function damage occurs, the NO yield and %FMD value will decrease. Generally, the lumen diameter method is used to determine the diameter of a vessel. However, since the subjects of this study were young, the tunica-intima tunica-media composite thickness was thin, and therefore difficult to determine.^(20)^ Instead, we estimated vessel diameter using the diameter between tunica externas for which clear imaging was obtained. The normal diameter measurement between lining membranes is 6% or more; however, using the diameter between tunica externas, the denominator and resting vessel diameter are large, and the %FMD value is determined to be low at about 1%.^(21)^

The %FMD value at each of the three postprandial times was indicated by lumen the rate of change from the preprandial value at 0 min since the preprandial %FMD value varies between individuals.

### Statistical analysis

The distribution of basic characteristic values, hematometry values, urine analysis values, %FMD values, and the %FMD rate of change was tested using the Shapiro-Wilk test. Verification of the differences between the two meals were determined following quantitative datum using the *t* test or Mann-Whitney *U* test for variables with a normal distribution. Results for items with a normal distribution are shown as mean ± SD, and/or median (minimum-maximum). We used IBM SPSS Statistics ver. 21.0 for all data analyses and statistical significance was set at less than 5%.

## Results

### Preprandial characteristics of subjects

The basic characteristics of the subjects (*n* = 6) before consuming the first test meal are shown in Table 1. The results of hematometry and %FMD measurements are shown in Table 3. Almost all hematometry values were within the normal range. The urine analysis results are shown in Table 4. All urine analysis values were within the normal range. No significant differences in any of these factors were seen before consuming the high organic P and the high inorganic P test meals.

### Influence on phosphorus metabolism

**Fig. 2:**
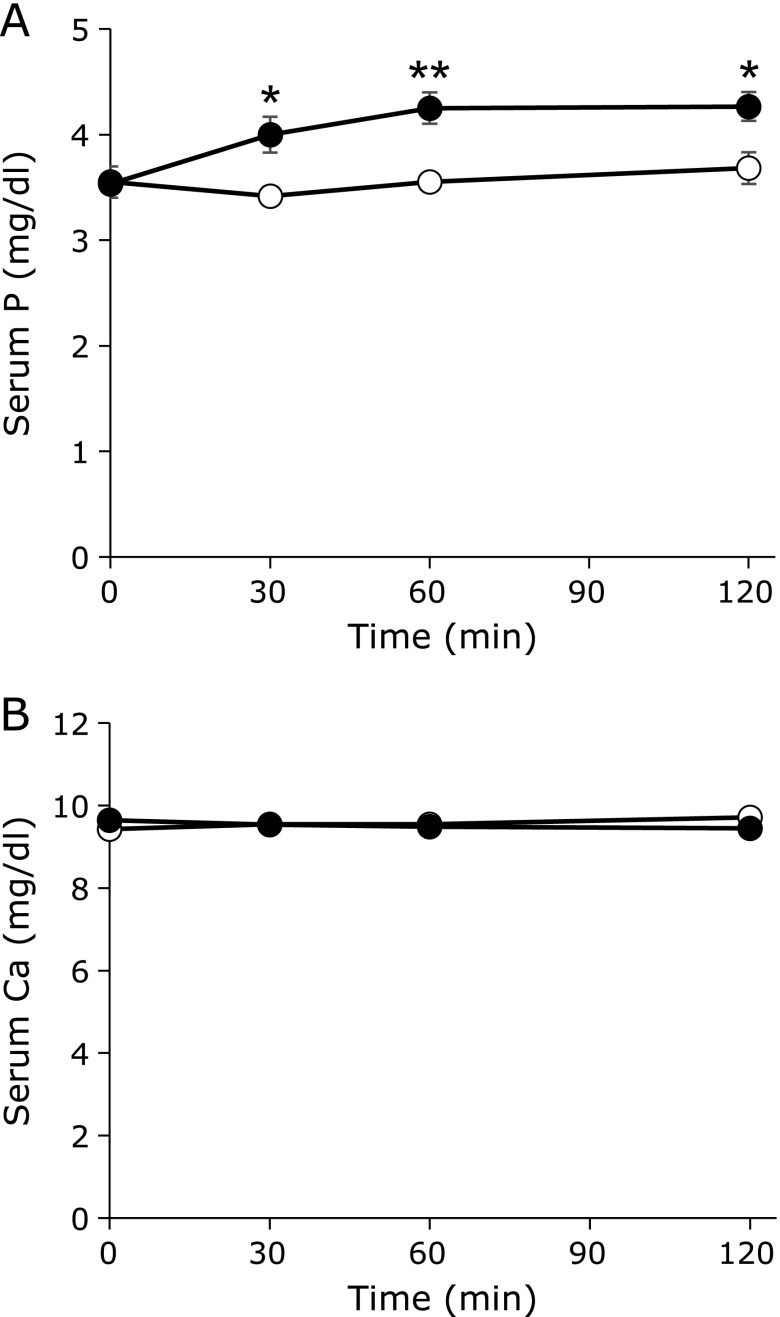
Changes in serum P (A) and serum Ca (B) over time. The white circles indicate the high organic P meal and the black circles indicate the high inorganic P meal. *p<0.05, **p<0.01 (*n* = 6).

As for HDL-C, LDL-C, the LDL/HDL ratio, TG, UA, Na, Cl, K and blood glucose level, no significant difference was seen between the two test meals at any of the time points. Changes in serum P, Ca and ionized Ca over time are shown in Fig. 2. Serum P significantly increased after the high inorganic P meal at 30, 60 and 120 min postprandial compared with preprandial (*p*<0.05, *p*<0.01 and *p*<0.001, respectively). On the other hand, a significant postprandial change was not seen after the high organic P meal. When comparing the two test meals, the high inorganic P meal resulted in higher serum P at all time points than the high organic P meal. There were no significant changes in serum Ca and ionized Ca (data not shown) at any of the postprandial time points with either of the two test meals, and there were no significant differences between the two test meals.

**Fig. 3:**
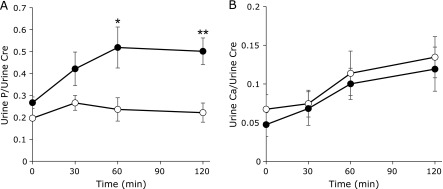
Changes in urine P/creatinine (A) and urine Ca/creatinine (B) over time. The white circles indicate the high organic P meal and the black circles indicate the high inorganic P meal. *p<0.05, **p<0.01 (*n* = 6).

Changes in urine P and Ca over time are shown in Fig. 3. Urine P showed a higher level of significance after the high inorganic P meal compared with the high organic P meal 60 and 120 min postprandial. Urine Ca showed an upward tendency postprandial after both test meals, but the difference was not significant.

**Fig. 4:**
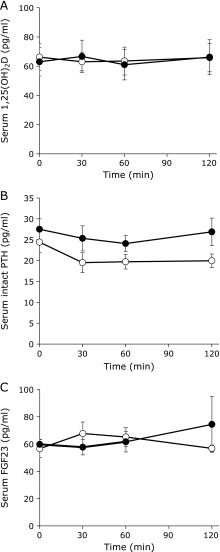
Changes in the P metabolism regulating hormones serum 1,25(OH)_2_D (A), serum intact PTH (B) and serum FGF23 (C) over time. The white circles indicate the high organic P meal and the black circles indicate the high inorganic P meal (*n* = 6).

Changes in phosphate metabolism regulation hormones in the blood over time are shown in Fig. 4. The 1,25(OH)_2_D, intact PTH and FGF23 levels did not show any significant differences between the two test meals at any of the time points.

### Effect on vascular-endothelium function

**Fig. 5:**
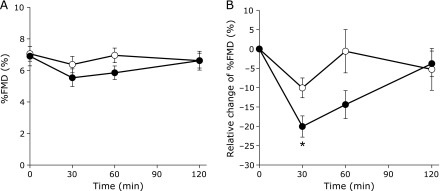
Influence of organic and inorganic P meals on endothelial function over time. Time course of %FMD (A) and relative change of %FMD (B) from preprandial. The white circles indicate the high organic P meal and the black circles indicate the high inorganic P meal. *p<0.05 (*n* = 6).

Changes in %FMD values over time are shown in Fig. 5. %FMD values showed reductions after both test meals at 30 min postprandial. After 30 min postprandial, %FMD values gradually rose and had mostly recovered to preprandial levels by 120 min postprandial (Fig. 5A). After the high organic P meal, a significant reduction was seen at 30 min postprandial compared with preprandial (*p*<0.05). However, %FMD values after the high inorganic P meal showed significant reductions at 30 and 60 min postprandial (*p*<0.01 and *p*<0.05, respectively). A tendency for %FMD to remain reduced was observed after the high inorganic P meal compared with the high organic P meal.

Furthermore, since individual differences were seen at preprandial, postprandial %FMD values are shown as the %FMD rate of change (Fig. 5B). The rate of change after the high inorganic P meal at 30 min postprandial (–20.0%) was significantly lower than that of the high organic P meal (–10.1%) (*p* = 0.024).

## Discussion

We investigated the influence of two different forms of P on vascular-endothelium function and found that high inorganic P meals resulted in elevated serum and urine P levels, and reduced %FMD values compared with high organic P meals. Therefore, it became clear that inorganic P has a stronger influence on vascular-endothelium function than organic P in healthy young men.

A reduction in vascular-endothelium function is one of the initial complications of arterial sclerosis. Factors such as diet, age, level of physical activity, and smoking have been reported to influence vascular-endothelium function.^(22,23)^ As for dietary factors, a high sugar foods, and high salinity foods, high fat diet have been reported to reduce vascular-endothelium function.^(24–26)^ These dietary factors increase oxidative stress and lead to vascular endothelial cell damage, through the activity reduction of endothelial NO synthase (eNOS) by a reactive oxygen species.

On the other hand, it has been reported that vascular-endothelium function is improved by the uptake of polyphenolics, such as a cocoa flavonol and curcumin.^(27,28)^ Polyphenolics have antioxidant properties and are known to control the reduction of vascular-endothelium function by reducing oxidative stress. Thus, vascular-endothelium function is subject to the influence of temporary sensitivity to dietary factors. The accumulation of these effects is considered to be related to the development or prevention of arterial sclerosis.

One study of dietary P and vascular-endothelium function in healthy men showed elevated serum P levels and reduced %FMD values after the intake of meals high in inorganic P.^(17)^ Furthermore, it has been shown that serum P levels and %FMD values have a negative correlation. In accordance with the elevation of serum P, oxidative stress increases, NO yield falls, and inorganic P stress is caused by damage to the vascular-endothelium function being carried out. Furthermore, it was assumed that inorganic P reduced %FMD values even though the amount of P loading was equivalent because the rate of absorption of inorganic P is higher than that of organic P.

One of the strengths of this study is that it shows the influence of two different forms of P on the reduction of vascular-endothelium function for the first time. It is thought that the regulatory mechanism through urinary excretion functioned because an elevation in urine P level was shown with an elevation in serum P level. Moreover, the alteration of phosphate metabolism in this study was temporary, so it did not influence P regulation hormones.

One limitation of this study is that there were few subjects. Although there were many subjects at the start of the study, those with serum P, FGF23 and %FMD values over the standard level were excluded. The subjects were also healthy young men, so the fact that many of them showed values exceeding the standards may reflect excessive P uptake in the current Japanese society.

In dialysis patients, hyperphosphatemia can induce vascular-endothelium damage. It is suggested that the long-term effects of this damage influence to arterial sclerosis, vascular calcification, and the development of CVD. In this study, the elevation of serum P after high inorganic P meal was normal range, and the reduction of %FMD had for the most part recovered by 120 min postprandial. However, it is thought that a habitual overabundant intake of inorganic P may cause dysfunction of the vascular endothelium in healthy persons by repeating the transient damage to the endothelial cells.

It has been reported that a reduction in renal function and a reduction in %FMD values are related, and dialysis patients have low %FMD values compared with healthy persons.^(29,30)^ It has been also reported that %FMD is decreased by indoxyl sulfate, a protein-bound uremic toxin, and leptin, which is elevated in CKD patients.^(31,32)^ Thus, P is one of the factors relevant to CVD, and this study showed that P, notably inorganic P, management in the diet is important. However, there are reports that only 17.8% of non-dialysis CKD patients in Japan did not know that inorganic phosphorus was contained in processed foods or drinks.^(33)^ In addition, 68.9% of patients did not know that excessive intake of phosphorus would harm. 37% of CKD patients who begin dialysis already have cardiovascular events.^(34)^ Thus, education about phosphorus intake is necessary form the early stage of CKD patients. The amounts of phosphorus in nutrition facts label of foods, in particular processed foods, is considered to be needed for patients to select foods.

In this study, it became evident that inorganic P contained in food additives has a strong effect on vascular-endothelium function and contributes to the development of CVD. Therefore, it is recommended that excessive intake of processed foods with high amounts of inorganic P be avoided. Further study of the factors that prevent the impact of inorganic P will lead to improvements in dietary management for dialysis patients and prevention of CVD.

## Figures and Tables

**Table 1:** Baseline characteristics of the study subjects

Characteristic	Mean ± SE
Age (year)	20.3 ± 0.4
Height (cm)	172.5 ± 2.8
Weight (kg)	61.8 ± 3.1
Percent of body fat (%)	14.6 ± 1.6
Lean body mass (kg)	52.6 ± 2.0
BMI (kg/m^2^)	20.7 ± 0.6
Systolic blood pressure (mmHg)	104.3 ± 1.0
Diastolic blood pressure (mmHg)	59.5 ± 3.0
Pulse (bpm)	74.2 ± 5.4

**Table 2:** Composition of test meals

	Weight (g)	Energy (kcal)	Protein (g)	Fat (g)	Carbohydrate (g)	P (mg)	Ca (mg)
High organic P meal							
Brown rice	130	455	8.8	3.5	95.9	377	12
Dried egg yolk	23	167	7.0	14.5	0.0	230	64
Non-fat dried milk	35	126	11.9	0.4	18.7	350	385
Seasoned laver	10	18	4.0	0.4	4.2	71	17
Gelation agent	30	99	0.0	0.1	27.4	174	12
Total		864	31.7	18.7	146.2	1,202	490

High inorganic P meal							

Milled rice	130	463	7.9	1.2	100.2	122	7
Dried egg yolk	5	36	1.5	3.1	0.0	50	14
Dried egg albumen	25	95	21.6	0.1	0.1	28	15
Salad oil	29	267	0.0	29.0	0.0	0	0
Salt	1	0	0.0	0.0	0.0	0	0
Additive P	1	0	0.0	0.0	0.0	1,000	0
Additive Ca	0.5	0	0.0	0.0	0.0	0	454

Total		861	31.1	33.4	100.3	1,200	490

**Table 3:** Preprandial serum chemistry and %FMD findings

	Normal range	High organic P meal	High inorganic P meal	*p*
%FMD	6≤	7.1 ± 0.5	6.9 ± 0.6	
UA (mg/dl)	3.6–7.0	6.1 ± 0.3	5.9 ± 0.2	0.569
Glucose (mg/dl)	<100	93.2 ± 1.5	88.5 ± 1.8	0.080
TG (mg/dl)	40–149	135.8 ± 29	101.8 ± 10.0	0.688
HDL-Cho (mg/dl)	41–86	56.3 ± 4.7	60 ± 5.5	0.623
LDL-Cho (mg/dl)	70–139	100.0 (67–183)	107.5 (87–182)	0.200
LDL/HDL ratio		1.64 (1.02–4.58)	1.67 (1.33–4.33)	1.000
Na (mEq/L)	135–150	140.3 ± 0.8	141.7 ± 0.3	0.169
K (mEq/L)	3.3–4.8	4.7 ± 0.3	4.5 ± 0.1	0.636
Cl (mEq/L)	98–108	101.7 ± 0.7	102.0 (97–103)	0.886
Ca (mg/dl)	8.4–10.2	9.4 ± 0.1	9.7 ± 0.1	0.171
Ionized Ca (mEq/L)	2.41–2.72	2.48 ± 0	2.52 ± 0	0.469
P (mg/dl)	2.5–4.5	3.6 ± 0.2	3.5 ± 0.1	0.926
intact PTH (pg/ml)	15.0–70.0	24.4 ± 2.5	27.5 ± 2.5	0.399
1,25(OH)_2_D (pg/ml)	20–60	66.2 ± 9.2	63 ± 9.7	0.983
FGF23 (pg/ml)	10–50	56.6 ± 6.6	59.8 ± 3.8	0.708

Values are mean ± SE or median (minimum–maximum).

**Table 4:** Preprandial urinary chemistry findings

	High organic P meal	High inorganic P meal
Cre (mg/dl)	257.8 (228.2–585.5)	241.7 ± 54.0
Ca (mg/dl)	14.4 ± 3.5	6.0 (2.9–36.0)
Ca/Cre	0.07 ± 0.02	0.05 ± 0.02
P (mg/dl)	50.3 (43.0–112.5)	64.3 ± 13.9
P/Cre	0.20 (0.16–0.21)	0.24 (0.22–0.39)
Urinary volume (ml)	48.2 ± 10.0	80.0 (30.0–440.0)
pH	5.9 ± 0.3	6.3 ± 0.4
Protein	(−)	(−)
Glucose	(−)	(−)
Urobilinogen	(±)	(±)
Occult blood	(−)	(−)
Bilirubin	(−)	(−)
Ketone body	(−)	(−)

Values are mean ± SE or median (minimum-maximum).

## Abbreviations

BMI: body mass index
Ca: calcium
CKD: chronic kidney disease
Cl: chloride
Cre: creatinine
CVD: cardiovascular disease
FGF23: fibroblast growth factor 23
FMD: flow mediated dilation
HDL-C: high density lipoprotein cholesterol
K: potassium
LDL-C: low density lipoprotein cholesterol
Na: sodium
NO: nitric oxide
P: phosphorus
PTH: parathyroid hormone
TG: triglyceride
UA: uric acid
